# Kidney Injury in Children after Hematopoietic Stem Cell Transplant

**DOI:** 10.3390/curroncol30030253

**Published:** 2023-03-13

**Authors:** Vinson James, Joseph Angelo, Lama Elbahlawan

**Affiliations:** 1 Division of Pediatric Nephrology, Department of Pediatrics, LeBonheur Children’s Hospital, Memphis, TN 38105, USA; 2 Division of Pediatric Nephrology, Department of Pediatrics, Baylor College of Medicine/Texas Children’s Hospital, Houston, TX 77030, USA; 3 Division of Critical Care Medicine, St. Jude Children’s Research Hospital, Memphis, TN 38105, USA

**Keywords:** hematopoietic cell transplant, kidney injury, renal replacement therapy, thrombotic microangiopathy, continuous kidney replacement therapy, fluid overload

## Abstract

Hematopoietic cell transplant (HCT), used for treatment of many malignant and non-malignant pediatric diseases, is associated with serious complications, limiting this therapy’s benefit. Acute kidney injury (AKI), seen often after HCT, can occur at different stages of the transplant process and contributes to morbidity and mortality after HCT. The etiology of AKI is often multifactorial, including kidney hypoperfusion, nephrotoxicity from immunosuppressive and antimicrobial agents, and other transplant-related complications such as transplant-associated thrombotic microangiopathy and sinusoidal obstructive syndrome. Early recognition of AKI is crucial to prevent further AKI and associated complications. Initial management includes identifying the etiology of AKI, preventing further kidney hypoperfusion, adjusting nephrotoxic medications, and preventing fluid overload. Some patients will require further support with kidney replacement therapy to manage fluid overload and AKI. Biomarkers of AKI, such as neutrophil gelatinase-associated lipocalin can aid in detecting AKI before a rise in serum creatinine, allowing earlier intervention. Long-term kidney dysfunction is also prominent in this population. Therefore, long-term follow-up and monitoring of renal function (glomerular filtration rate, microalbuminuria) is required along with management of hypertension, which can contribute to chronic kidney disease.

## 1. Acute Kidney Injury

AKI is a relatively common complication after HCT, with a reported incidence rate of 21–84% [1,2]. Table 1 summarizes published studies reporting AKI in the pediatric HCT population [3,4,5,6,7,8,9,10]. In a metanalysis including 571 children post HCT, AKI developed in 124 (21.7%) within the first 100 days post-transplant, with a median onset of 4–6 weeks [11]. In a cohort of 408 adult patients post HCT, AKI was observed in 64% within 100 days post-transplant, but most had mild AKI (62% had stage 1 AKI). More importantly, worse outcome was observed in patients with AKI. Compared to patients without AKI, patients in whom AKI developed had inferior 2-year overall survival and higher incidence of grade 3–4 acute graft versus host disease (GVHD) [12]. 

**Figure 1:**
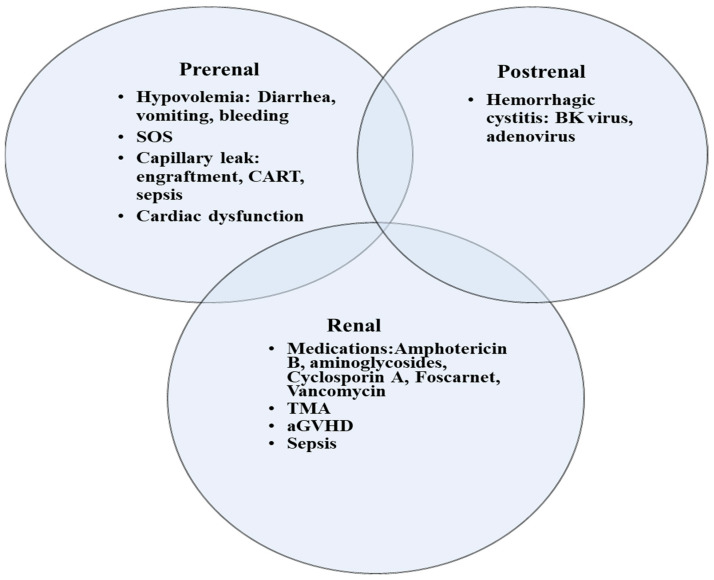
Etiologies of AKI in children following HCT. SOS, sinusoidal obstructive syndrome; TMA, thrombotic microangiopathy; aGVHD, acute graft versus host disease; CART, Chimeric antigen receptor (CAR) T-cell therapy.

Common risk factors for kidney injury include myeloablative conditioning, older age, acute GVHD, and SOS (Table 1). Satwani et al. observed a significant increase in the incidence of kidney injury in children who received myeloablative conditioning versus reduced intensity conditioning (45.7% and 17.1% respectively) [6]. In addition to its contribution to a higher rate of mortality, previous AKI predisposes patients to chronic kidney disease (CKD). In a cohort of 158 adult allogeneic HCT survivors, the risk of CKD ≥ stage 3 was approximately 10-fold higher in patients in whom AKI developed following HCT [13]. AKI is encountered at any stage in the transplant process, although often at the earlier stages. Early in the pre-transplant phase, many children receive myeloablative conditioning regimen with or without total body irradiation (TBI) that can induce kidney injury. Shortly after transplant, nephrotoxic immunosuppressive medications such as calcineurin inhibitors are given to mitigate the risk of GVHD. In addition, children are at a higher risk of infection and subsequent sepsis due to their immune-compromised status. In many instances, the etiology of AKI is multifactorial and includes hypoperfusion in the setting of capillary leak or sepsis resulting in acute tubular necrosis, drug induced nephrotoxicity, thrombotic microangiopathy (TMA), and sinusoidal obstruction syndrome (SOS) (Figure 1) [1]. Drug induced nephrotoxicity is relatively common in HCT patients. Antimicrobials that are often used to treat infections post HCT such as aminoglycoside, vancomycin, or amphotericin can induce direct kidney injury. Nephrotoxicity is also encountered with calcineurin inhibitors such as cyclosporin or tacrolimus that can cause kidney arteriolar vasoconstriction via activation of the renin–angiotensin–aldosterone system [14]. In addition, calcineurin inhibitors may trigger endothelial injury and subsequent TMA [15]. Moreover, kidneys can be a target of GVHD, although less described than other organs like skin, liver, and lungs. Kidney injury related to GVHD is mediated by donor T-cells as well as proinflammatory cytokines. Kidney GVHD can present as AKI, nephrotic syndrome, glomerulonephritis, and TMA [16]. However, the most common presentation is nephrotic syndrome with a high degree of proteinuria, hypoalbuminemia, and edema. Hemorrhagic cystitis can cause obstructive kidney injury when clots in the bladder obstruct the outflow tract. The etiology of hemorrhagic cystitis is usually multifactorial, but often encountered with the use of cyclophosphamide or in the context of reactivation of virus infections such as BK virus, adenovirus, and cytomegalovirus. Treatment include hyperhydration, diuresis, and bladder irrigation with a three-way bladder catheter.

## 2. Criteria for AKI Diagnosis and Staging

One reason that the incidence of AKI varies so widely (21–84%) is that non-standardized definitions of AKI were used in prior studies. More standardized and widely used AKI scoring systems have now been developed, the most recent and commonly used of which are the KDIGO guidelines. Diagnosis and staging of AKI relies mainly on two factors: serum creatinine (sCr) level and urine output (Table 2). However, many factors affect sCr level, such as muscle mass and age, leading to over- or under-diagnosis of AKI. Volume status also influences creatinine level; for example, fluid overload, which is relatively common in children with AKI post HCT, results in an erroneous lower sCr value, leading to underestimation of the degree of AKI and a delay in diagnosis. Although methods of correcting sCr for particular clinical scenarios have been proposed, such as the following formula for correcting sCr for fluid overload: Corrected cr = sCr × [1 + Net fluid balance/Total body water] where total body water (TBW) = 0.6 × weight (kg), sCr can still have some limitations in accurately assessing eGFR [17]. 

## 3. Special Disease Conditions Post HCT That Are Associated with AKI

### 3.1. Transplant-Associated Thrombotic Microangiopathy

Transplant-associated thrombotic microangiopathy (TA-TMA) is a life-threatening complication that is encountered early in the post-HCT phase. The incidence of TA-TMA in children is 16%, with a median onset of 47 days post-transplant [18]. Risk factors for TA-TMA include acute GVHD, infectious process (especially viral), mismatched donor, multiple HCTs, and myeloablative conditioning [18]. The pathophysiology of TA-TMA involves an initial endothelial injury triggered by factors such as chemotherapy or infection that results in an increase in the proinflammatory cytokines, procoagulant factors, and soluble adhesion molecules. This combination promotes further endothelial injury and initiates and propagates the complement cascade, resulting in platelet aggregation, fibrin deposition, and microthrombi formation. The laboratory characteristics of TA-TMA include elevated lactated dehydrogenase, non-immune mediated hemolytic anemia, presence of schistocytes on the peripheral blood smear, thrombocytopenia, proteinuria, and elevated plasma sC5b-9 (≥244 ng/mL). Renal involvement with microangiopathy is common in patients with TA-TMA. Renal histopathology findings include fibrin deposition in the glomeruli, narrowing of the capillary lumen, presence of fragmented red blood cells, basement membrane duplication, and edema of the endothelium [19]. In a cohort of 98 children in whom TA-TMA developed post HCT, AKI developed in 66% of the cohort, and 15% required renal replacement therapy [20]. Hypertension is one of the earliest manifestations in these patients, occurring as early as 14 days before the diagnosis of TA-TMA [21]. Moreover, proteinuria is common and present in 80% of children with TA-TMA. Proteinuria is an important indicator of renal dysfunction that presents early during TA-TMA and is a marker of severe disease. It is associated with a higher 6-month mortality rate (27% in patients with proteinuria vs 5% in patients without proteinuria [*p* = 0.04]) [20]. Additionally, the overall survival rate of children with TA-TMA (78%; 76/98) is significantly lower than that of those without TA-TMA (93%; 490/516) (*p* = 0.001) [20].

Eculizumab is a monoclonal antibody against the complement component C5, which blocks the formation of the membrane attack complex (MAC or C5b-9) and thus prevents endothelial damage. In a cohort of 64 pediatric HCT patients with high-risk TA-TMA and multiorgan dysfunction, the survival rate improved dramatically with the use of eculizumab (66% in 1-year post HCT in treated vs 16.7% in a previously reported untreated cohort) [22]. Notably, 70% of survivors still had proteinuria on long-term follow-up, and their best cystatin C estimated glomerular filtration rate (eGFR) after recovery from TA-TMA was still lower than their pre-transplant baseline value [22].

### 3.2. Sinusoidal Obstruction Syndrome

Sinusoidal obstruction syndrome (SOS) is associated with multiorgan dysfunction and a high mortality rate [23]. SOS occurs in the early stage post HCT secondary to cytotoxic therapy or radiotherapy [24]. The incidence rate is 20–60%. Diagnosis of SOS is based on the following criteria (two or more criteria present) [25]:

- Consumptive and transfusion-refractory thrombocytopenia;
- Weight gain on 3 consecutive days despite the use of diuretics, or a weight gain of >5% above baseline weight within 72 h;
- Increase in bilirubin from baseline on 3 consecutive days, or bilirubin ≥ 2 mg/dL within 72 h;
- Hepatomegaly (best if supported by imaging) above baseline value;
- Ascites (best if supported by imaging) above baseline.

Kidney injury in SOS is attributed to hypoperfusion and vasoconstriction and is associated with fluid overload. Managing the kidney injury requires fluid restriction and use of diuretics to reduce fluid overload. Renal replacement therapy may be necessary if fluid overload persists, and urine output remains inadequate despite diuretic treatment [26]. In such cases, continuous kidney replacement therapy (CKRT) is typically the preferred modality, enabling more controlled and dynamic adjustment of fluid removal, particularly in hemodynamically unstable patients. Raina et al. described the use of CKRT in six children who had SOS post HCT: Four survived (mortality rate = 34%), and one survivor experienced end-stage renal disease and kidney transplantation [27]. Defibrotide, the drug of choice for treatment of SOS, reduces the mortality rate and reverses organ dysfunction [28].

### 3.3. Fluid Overload

Fluid overload (FO) is common in critically ill children and negatively affects outcome [29,30]. Additionally, FO can exacerbate kidney injury by worsening kidney venous hypertension, impairing perfusion pressure capacity of the glomerular capillaries. Cumulative fluid balance is often used interchangeably with fluid overload and is calculated as follows: Fluid intake – Fluid output (L)/ICU admission weight (kg) × 100 [31]. FO can also be calculated by comparing current weight to admission weight if fluid balance information is not available. FO > 10% is common in critically ill children and was observed in 33% of a large cohort of 1017 critically ill children [29]. FO was associated with higher risk of mortality, kidney adverse events, and increased duration of mechanical ventilation (MV) and ICU stay. A metanalysis including 44 pediatric studies showed a 6% increase in odds of mortality with each 1% increase in FO [30]. The adverse effect of FO is also prevalent in the pediatric HCT population [32,33]. Children with FO > 10% at CKRT initiation were 6.16 times more likely to die than those with FO ≤ 10% in a cohort of 68 critically ill children with cancer and post HCT (23 patients). In mechanically ventilated pediatric HCT patients, higher cumulative fluid balance is associated with an increased mortality rate [33,34]. In a cohort of 198 children with acute respiratory failure, a 3% increase in ICU mortality occurred for every 1% increase in the cumulative fluid balance on day 3 of the course of mechanical ventilation (adjusted odds ratio = 1.03; 95% CI, 1.00–1.07) [34].

FO is a new post-HCT toxicity category with the following proposed grading system [1]:

Grade 1: AKI Stage 0 or 1; fluid overload < 10%

Grade 2: AKI Stage 0 or 1; fluid overload > 10%

Grade 3: AKI Stage 2 or 3; fluid overload < 10%

Grade 4: AKI Stage 2 or 3; fluid overload > 10%

Close monitoring of daily fluid balance and weight is crucial to prevent FO. This allows for early recognition and prompt management of FO to reduce further kidney injury and improve survival. Unfortunately, even significant FO is often missed. Al-Lawati et al. found that clinicians did not recognize FO > 15% in 30% of pediatric patients [35]. In addition to concentrating the volume of the administered medications, diuretics such as furosemide or bumetanide are administered either intermittently or as a continuous infusion. CKRT may be considered when FO is > 10% if diuretic therapy fails to achieve euvolemia.

### 3.4. CAR T-Cell Therapy

Chimeric antigen receptor (CAR) T-cell therapy, used for treatment of hematologic malignancies, involves the utilization of engineered cytotoxic T-cell to recognize specific tumor antigen. AKI occurs with this therapy secondary to cytokine release syndrome (CRS), a well described complication of this therapy which can lead to organ dysfunction. Hypoperfusion secondary to capillary leak and proinflammatory cytokines contribute to AKI encountered post CAR-T cell therapy. AKI is usually mild in these cases [36]. In a cohort of 39 children with acute lymphoblastic leukemia treated with an anti-CD19 CAR T-cell therapy, 46% developed AKI with grade 3–4 CRS although none of the patients required CKRT [37]. Similarly, in adults, the reported cumulative incidence of AKI by day 100 was 30% in patients with non-Hodgkin lymphoma and none required CKRT [36]. Prompt management of serious CRS toxicity is important to ameliorate the inflammatory response and reduce organ dysfunction. Such management includes anti-cytokine therapy, such as tocilizumab, and corticosteroids [38].

## 4. Continuous Kidney Replacement Therapy

Nearly one-third of patients with AKI require kidney replacement therapy (KRT) [1]. CKRT is used often in the ICU to deliver KRT because it is tolerated better than intermittent hemodialysis (IHD) in hemodynamically unstable critically ill children. During CKRT, fluid removal and solute clearance occur continuously, promoting better control of fluid status. Solute clearance occurs by either convection, diffusion, or both, whereas fluid is removed via ultrafiltration. Hemofiltration modes of CKRT can increase removal of small and medium-sized solutes by convection (solute drag); in contrast, hemodialysis modes mainly remove small-sized molecules by diffusion (concentration gradient). Flores et al. reported better survival in children post HCT (51 patients) with convective modes of CKRT than with dialytic mode [39]. However, mortality was similar using the hemofiltration or the hemodialysis modality in a metanalysis including 19 randomized trials in patients with AKI [40]. 

No consensus exists on the optimal time for initiation of CKRT and whether early initiation can improve outcome. Most evidence is from adult randomized trials that compared the early initiation of CKRT to using a standard strategy. One of the largest adult trials, the STARRT-AKI trial, randomized 3019 critically ill adults with AKI to either an accelerated RRT strategy (initiated within 12 h in adult critically ill patients with Stage 2 or Stage 3 AKI) or a standard strategy. The accelerated RRT strategy did not reduce mortality compared to the standard strategy, and survivors of the accelerated RRT strategy had a higher risk of adverse events and dependence on kidney replacement therapy [41]. In contrast, in the ELAIN trial that included 231 critically ill patients with AKI, a lower mortality in the early RRT group compared to the delayed initiation group was observed (39% versus 54% respectively) [42]. A recent metanalysis that had 5193 critically ill patients with AKI did not demonstrate improved survival or recovery of renal function using the early RRT initiation strategy rather than later RRT [43]. Although the evidence does not support early initiation of KRT in the context of AKI, ample evidence in the literature supports initiating CKRT at the earlier stage of FO to reduce mortality. CKRT should be considered in children with FO > 10%, especially when associated with pulmonary edema or worsening AKI. 

The recommended dose of CKRT is 20–25 mL/kg/h, or 2000 mL/1.73 m^2^/h in children. However, the delivered dose is usually less than prescribed dose due to interruptions that occur during CKRT, such as circuit clotting, scheduled filter changes, and pauses for other procedures. Thus, KDIGO recommends a 25–30 mL/kg/h prescribed dose to achieve a delivered dose of 20–25 mL/kg/h [44]. A CKRT dose > 35 mL/kg/h is not recommended as it does not reduce mortality [45]. Anticoagulation is crucial to prevent circuit clotting and extend the circuit lifespan, thus minimizing treatment interruptions. Systemic heparin infusion or regional citrate anticoagulation (RCA) can be used for anticoagulation. In a cohort of 638 critically ill adults on CRRT, median filter life span was significantly higher with RCA than heparin (47 h vs. 26 h). Bleeding complications were also significantly less in the citrate group than in the heparin group (5.1% vs. 16.9%) [46]. Therefore, RCA may be a better option for anticoagulation than systemic heparin in the HCT population that is at higher risk of bleeding due to thrombocytopenia or coagulopathy.

## 5. Transition from CKRT to IHD/Discontinuation of CKRT

The optimal timing for successful discontinuation of CKRT or switch to IHD is difficult to predict. Renal recovery is usually preceded by an increase in urine output. Urine output > 500 cc/day is used in some adult patients as a criterion to discontinue KRT [47]. Factors that have been shown to predict successful liberation include the hourly urine output within 12 h before CKRT discontinuation, serum creatinine level within 24 h before liberation, and the cumulative fluid balance (from ICU admission to CKRT discontinuation) [48]. In general, children are switched from CKRT to IHD when FO is resolved and they are hemodynamically stable.

## 6. Outcomes of KRT

ICU mortality in children post HCT requiring CKRT is estimated to be 52–65% [2,49]. The 1-year overall survival rate is also poor (27.4% (95% CI: 16–40.5%, *p* < 0.0001)) [1]. Reported factors that are associated with mortality include FO > 10%, mechanical ventilation, vasoactive support, and neutropenia at the end of CKRT [2].

## 7. Biomarkers of AKI in Children with HCT

Given the potential shortcomings of sCr as a marker of AKI, several additional biomarkers of AKI have been developed and studied. These biomarkers measure either glomerular function or renal tubular damage and can aid in early detection of AKI (Table 3). 

### Cystatin C

Cystatin C (CysC) is a low molecular weight protein which is present in all nucleated cells and inhibits cysteine proteases. It is freely filtered by the glomerulus, then reabsorbed by the proximal tubule epithelium and catabolized. It is present in urine only in instances of tubular injury. Serum CysC levels can be altered by several factors such as corticosteroids, inflammatory status, and chemotherapy. Levels may also be elevated in patients with leukemia without any documented AKI secondary to increased cell turnover [1]. CysC can detect decline in renal function and GFR 24–48 h before serum creatinine levels. Furthermore, a higher CysC level at discontinuation of KRT was found to be an independent predictor of chronic dialysis [50].

## 8. Tubular Injury Markers 

Several tubular injury markers have been described and investigated for their utility in early recognition of AKI. Neutrophil gelatinase-associated lipocalin (NGAL) levels are elevated in the urine early after ischemic, septic, or toxic injury and precede the rise in serum creatinine levels by 48 h [51]. In addition, urine NGAL can differentiate intrinsic renal damage (where it is elevated) from prerenal acute injury related to hemodynamic alterations due to hypovolemia [52]. N-acetyl-beta-D-glycosaminidase (NAG) urine level is highly specific to tubular injury [53]. Kidney injury molecule-1 (KIM-1) participates in both kidney injury and healing processes [54]. In fact, urinary KIM-1 is highly sensitive and specific for drug-induced kidney injury.

Fatty acid-binding protein 1 is another early biomarker of AKI and can predict the need for dialysis [55]. An increased level of L-FABP at the time of ICU admission is associated with higher risk of AKI and mortality rate [56,57]. The NGAL and L-FABP combination can predict renal recovery after AKI (higher levels associated with non-recovery) [58]. Urinary IL-18 level can predict the initiation of RRT and mortality [59,60]. The combined product of tissue inhibitor of metalloproteinases-2 (TIMP-2) and insulin-like growth factor–binding protein-7 (IGFBP-7), expressed as [TIMP-2] [IGFBP7], predicts the risk of AKI as well as the need for CKRT and death in critically ill adults [61,62].

## 9. Chronic Kidney Disease

CKD in children post HCT has a reported incidence of 48%. CKD is defined by estimated GFR < 90 mL/min/1.73 BSA or presence of markers of kidney damage for >3 months [63]. CKD can develop as early as 6 months and up to 10 years following transplant. Incidence of CKD in this population is 10-fold higher than in the healthy population. In a cohort of 1635 adult and pediatric HCT patients, CKD developed in 23% [64]. 

The most common etiologies and risk factors for CKD development are TMA, total body irradiation, nephrotic syndrome, AKI, acute GVHD, and drug toxicity (calcineurin inhibitors) [65]. Estimated GFR at the time of AKI is an important risk factor for the development of CKD. In a cohort of 275 children post allogeneic HCT, CKD developed in 69.5% and 69.8% at 1 and 3 years if GFR was < 80 mL/min/1.73 m^2^ at the initial AKI episode [66]. Therefore, renal function in these higher-risk children who developed AKI with GFR <80 mL/min/1.73 m^2^ must be monitored closely for early detection of CKD. Similar to the approach to AKI noted previously, the management of CKD post HCT requires regular monitoring for long term changes in estimated GFR to identify patients with CKD, followed by preventive strategies that minimize nephrotoxic medication exposure, ensure adequate hydration and nutrition, optimize blood pressure control, and minimize proteinuria.

Albuminuria (albumin-to-creatinine ratio over 30 mg/g) is an important parameter to monitor in children post HCT during long-term follow-up for early recognition of CKD. Albuminuria is relatively common (detected in 50% of patients one-year post HCT) [67]. Furthermore, albuminuria at day 100 was associated with CKD at 1 year (OR = 4.0; 95% CI = 1.1 to 14.6). Proteinuria at day 100 conveyed a six-fold increase in the risk of non-relapse mortality by 1 year post HCT. Moreover, hypertension seen in 20–70% of patients post HCT can contribute to the development and progression of CKD [68]. Close monitoring for the development of hypertension is warranted for children post HCT and should follow similar guidelines for the detection and management of hypertension in children in other settings, including obtaining 24-h ambulatory blood pressure monitoring when available [69]. Angiotensin-converting enzyme inhibitors (ACEi) or angiotensin II receptor blockers (ARB) can be used to preserve kidney function, control hypertension, and reduce proteinuria [69,70]. Other antihypertensive medications that can be utilized for optimal control of blood pressure or if ACEi/ARB is not tolerated include long-acting calcium channel blockers and diuretics. In addition to the initiation of antihypertensive medications, other lifestyle modifications including low sodium diet and increased physical activity should be encouraged to reduce long term risk of hypertension. Target BP goals for patients with CKD should be less than or equal to 50th percentile for age, sex, and height unless achieving this is limited by symptomatic hypotension [69,71]. 

Additional long-term follow-up for HCT patients with CKD includes monitoring and treatment of other consequences of progressive CKD including electrolyte disturbances, such as metabolic acidosis, anemia of CKD, and CKD-related bone disease [65]. Consultation with nephrology is recommended for patients with any degree of CKD to coordinate CKD care, and frequency of follow up is then based on the severity of CKD. These multiple complications of CKD highlight the need for a multidisciplinary approach to post-HCT patients with CKD as these patients may have unique risk factors and needs related to the underlying oncologic diagnosis and post-HCT course to consider when managing their CKD.

## Figures and Tables

**Table 1:** Summary of studies of AKI in pediatric HCT patients.

Study	No. of Patients	AKI Criteria	Incidence of AKI	Risk Factors	Survival
Daraskevicius et al. [3]	51	pRIFLE	35.3%	Older age Higher BMI	
Koh et al. [4]	1057	AKIN	68.2% Stage 1: 22% Stage 2: 41.4% Stage 3: 36.6% KRT: 30.3% in patients with Stage 3	Older age Myeloablative conditioning Unrelated donor SOS aGVHD grade II-IV	1 year OS No AKI: 66.1% Stage 1: 73.4% Stage 2: 63.9% Stage 3: 47.3% Stage 3 requiring KRT: 7.5%
Kizilbash et al. [5]	205	pRIFLE	84% R: 71 (35%) I: 67 (33%) F/L/E without dialysis: 21 (10%) F/L/E with dialysis: 14 (7%)	Race: African American SOS	HR mortality F/L/E 8.41 (4.55–15.53)
Satwani et al. [6]	170	≥50% decrease in eCrCl from the baseline	33% at 30 d post HCT 55% at 3 m post HCT CKRT: 14.7%	Myeloablative conditioning	HR mortality: 2 with AKI at 1 m
Yu et al. [7]	96		29.2% Stage 1: 14.6% Stage 2: 12.5% Stage 3: 2.1%	aGVHD Myeloablative conditioning	Mortality Grade 1: 57.1% Grades 2–3: 78.6%
Ileri et al. [8]	57	AKIN	42% Stage 1: 21% Stage 2: 16% Stage 3: 5%	Cyclosporin A Amphotericin B SOS	Mortality: 21% within 100 d
Hazar et al. [9]	34		26.4%		Mortality: 39% within 6 m
Kist-van Holthe et al. [10]	66		21%	SOS Cyclosporin A Foscarnet	

AKIN, Acute Kidney Injury Network; KDIGO, Kidney Disease Improving Global Outcomes; pRIFLE, pediatric Risk, Injury, Failure, Loss, End-Stage Renal Disease; AKI, acute kidney injury; CKRT, continuous kidney replacement therapy; KRT, kidney replacement therapy; eCrCl, estimated creatinine clearance; aGVHD, acute graft versus host disease; SOS, sinusoidal obstruction syndrome; HR, hazard ratio.

**Table 2:** Staging criteria for AKI.

Stage	AKIN Staging	KDIGO Staging	pRIFLE Staging
**Stage 1**	Scr: 1.5–2.0x Bl OR ≥ 0.3 mg/dL 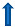	Scr:1.5–1.9x Bl OR ≥ 0.3 mg/dL 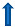	R = Risk for renal dysfunction eGFR by 25% 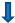
	UO: <0.5 mL/kg/h for >6 h	<0.5 mL/kg/h for 6–12 h	<0.5 mL/kg/h for 8 h
**Stage 2**	Scr: >2–3x Bl	Scr: 2.0–2.9x Bl	I = Injury to the kidney eGFR 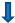 by 50%
	UO: <0.5 mL/kg/h for >12 h	<0.5 mL/kg/h for 12–24 h	<0.5 mL/kg/h for 16 h
**Stage 3**	Scr: >3x Bl OR KRT	Scr: 3.0x Bl OR ≥ 4.0 mg/dL OR KRT OR eGFR < 35 mL/min/1.73 m^2^	F = Failure of kidney function eGFR 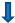 by 75% OR eGFR < 35 mL/min/1.73 m^2^
	UO: <0.3 mL/kg/h for 24 h OR Anuria for 12 h	<0.3 mL/kg/h for ≥24 h OR Anuria for ≥12 h	<0.3 mL/kg/h for 24 h OR Anuria for 12 h L = Loss of kidney function Persistent failure > 4 weeks E = End-stage renal disease Persistent failure > 3 months

AKIN, Acute Kidney Injury Network; KDIGO, Kidney Disease Improving Global Outcomes; pRIFLE, pediatric Risk, Injury, Failure, Loss, End-Stage Renal Disease; AKI, acute kidney injury; Scr, serum creatinine; Bl, baseline; UO, urine output; eGFR, estimated glomerular filtration rate; KRT, kidney replacement therapy.

**Table 3:** Biomarkers in AKI.

Biomarker	Characteristic	Detection Time	Peak	AUC for AKI Detection	Limitations
**Glomerular injury**					
Cystatin C	13-kDa protein that is present in all nucleated cells, protease inhibitor	2–48 h	6–8 h		Influenced by inflammation, muscle mass, and high-dose steroids
**Renal tubular injury**					
NGAL	25-kDa protein of the family of lipocalins with bacteriostatic function	2–24 h	6–12 h	0.8 (0.72–0.87)	False elevation in sepsis and malignancy
NAG	>130-kDa proximal tubular lysosomal enzyme	2–4 h		0.6	Elevated in diabetes and albuminuria
KIM 1	38.7-kDa type I transmembrane glycoprotein	1–24 h		0.85	Slow rise and non-specific May be elevated in the settings of chronic proteinuria and inflammatory diseases
Interleukin-18	24-kDa cytokine	4–48 h	12 h	0.75	Low sensitivity/specificity
L-FABP	14-kDa lipid binding protein	12–72 h			May lose its specificity when liver disease is present
TIMP 2	21-kDa protein, endogenous inhibitor of metalloproteinase activities, involved in G1 cycle arrest	1–12 h		0.8	Proteinuria interferes with the test results Elevated in diabetes
IGFBP7	29-kDa protein, IGF-1 receptor antagonist, involved in G1 cycle arrest			0.76	

NGAL, Neutrophil gelatinase-associated lipocalin; KIM-1, Kidney injury molecule-1; L-FABP, Liver type fatty acid binding protein; TIMP 2, Tissue inhibitor of metalloproteinase 2; IGFBP7, insulin-like growth factor-binding protein-7.
